# Influence of Sodium Hypochlorite and Chlorhexidine on the Dynamic Cyclic Fatigue Resistance of XP Endo Shaper Instruments

**DOI:** 10.1055/s-0041-1735934

**Published:** 2021-12-22

**Authors:** Alexandra Aparecida Tanomaru, Ana Grasiela Limoeiro, Adriana de Jesus Soares, Elson Lopes Medeiros Junior, Gabriel Rocha Campos, Sílvia Kaoru Hamasaki, Wayne Martins Nascimento, Luiz Meton Horta, Paula Avelar da Silva Ribeiro Goulart, Viviane Rangel do Couto, Patrícia Bastos Alves, Verônica Magalhães, Danilo De Luca Campos, Marcos Frozoni

**Affiliations:** 1 Department of Endodontics, São Leopoldo Mandic Dental Research Center, Campinas, São Paulo, Brazil; 2 Department of Endodontics, Ilhéus School of Dentistry, Ilhéus, Bahia, Brazil; 3 Department of Restorative Dentistry, Piracicaba Dental School, State University of Campinas- UNICAMP, Piracicaba, São Paulo, Brazil

**Keywords:** chlorhexidine digluconate, irrigation solutions, sodium hypochlorite, XP-Endo Shaper

## Abstract

**Objective** This study evaluated the dynamic cyclic fatigue resistance of the XP-Endo Shaper (XPS), associated with chlorhexidine digluconate (CHX) or sodium hypochlorite (NaOCl) in two different formulations: gel (G) or liquid (L).

**Materials and Methods** Sixty XPS were used in an artificial stainless-steel canal, and the files were fully immersed in the irrigating solution throughout the experiment until the fracture. The files were divided into six groups ( *n* = 10) based on the irrigation solution used: NaOCl(L), NaOCl(G), CHX(L), CHX(G), natrosol gel (NAT) (control), and lubricating oil (LO) (control). The artificial canal was manufactured 1.5 mm wide, 20 mm long, and, 3.5 mm deep with a straight cervical segment measuring 14.29 mm; an apical segment of 4.71 mm with 3 mm radius; and 90 degrees of curvature apical 1 mm long straight segment. Resistance to cyclic fatigue was determined by recording the number of cycles to fracture (NCF).

**Results** The CHX(G), CHX(L), and OIL (LO) groups showed no significant difference between them and presented longer time to fracture ( *p* > 0.05). NaOCl(L) shows the lowest NCF without significant differences between NaOCl(G) and NAT. The NCF of the NaOCl(G) was statistically similar to the CHX(L) and statistically lower than the CHX(G) and OIL groups. NAT did not present a statistical difference of the NaOCl(L), NaOCl(G), and presented a significantly lower NCF than the CHX(G) ( *p* < 0.01).

**Conclusion** The use of CHX(G) resulted in increased cyclic fatigue resistance of the XPS instruments compared to NaOCl or LO.

## Introduction

The development of new nickel-titanium (NiTi) alloy led to a higher success rate of root canal treatment, reducing clinical time and instrument fracture. 1 The XP-Endo Shaper (XPS; FKG, La Chaux-de-Fonds, Switzerland) plays a leading role in chemomechanical preparation and root canal disinfection. It is made of MaxWire alloy 2 ; offers high flexibility and fatigue resistance; and can penetrate the canals easily and quickly, expanding or contracting, increasing canal volume, surface area, percentage of touched walls, and the amount of dentin removed. 3 This single file system is associated with a lower frequency of postoperative pain compared to multi-instrument files, 4 is effective in bacterial reduction of oval root canals with necrotic pulps, 4 and is also indicated for use in curved canals as it has the ability to maintain the original shape with minimal transport. 5

However, endodontic files are unable to reach all root canal walls, 6 chemical substances assume a fundamental role in acting in these places where the instrument cannot reach. 7 Of all the substances currently used for root canal irrigation, sodium hypochlorite (NaOCl) seems ideal because it meets more requirements than any other known irrigating solution. 8 9 The most used formulation of NaOCl is liquid NaOCl (L). However, it is potentially toxic in its liquid formulation when in contact with periapical tissues. 10 The sodium hypochlorite gel form NaOCl(G) reduces the risk of debris extrusion into periapical tissues, 11 and it is effective in reducing *Enterococcus faecalis* biofilm, but this effect is less than that of NaOCl liquid. 12

Chlorhexidine digluconate (CHX) is an option for endodontic irrigants that could replace hypochlorite with some limitation. 13 CHX is a cationic compound with excellent antibacterial properties. 14 It has shown antimicrobial activity against both forms of intracanal bacterial growth (planktonic and biofilm bacteria), 13 and its contact with vital tissues presents low toxicity. 13 For root canal chemomechanical preparation, CHX can be used in a liquid CHX(L) or a gel CHX(G) presentation. CHX(G) consists of a gel base (natrosol, a hydroxyethyl cellulose; pH = 6–9) and chlorhexidine gluconate. 15 CHX(G) formulation can perfectly replace the CHX(L), improving the reduction of smear layer formation, compensating for its incapacity to dissolve organic tissues. It has a better residual effect due to its substantivity (up to 24 hours). 15 The gel formulation may keep the active principle of CHX in contact with the microorganisms for a longer time, inhibiting their growth. 16 A brown precipitate is formed by mixing NaOCl and CHX. 17

Despite technological innovations in endodontics, fractures from mechanized NiTi instruments still occur in two ways: torsional or cyclic fracture. 18 Therefore, the present study aimed to evaluate the dynamic cyclic fatigue resistance of the XPS files in association with two solutions in different formulations: NaOCl(L), NaOCl(G), CHX(L), and CHX(G). The null hypothesis was that the different irrigants do not influence the XPS files' dynamic cyclic fatigue resistance at body temperature.

## Materials and Methods

The sample size and power for statistical testing were calculated by analyzing variance using the software G*Power 3.1.9.4. For the effect size of 0.571, obtained from a pilot study ( *n* = 3), the significance level of 5%, and power of 90%, the sample calculation indicated the need for 60 files ( *n* = 10) in the present study.

Sixty 25-mm long XPS instruments were equally assigned to six groups ( *n* = 10) based on the irrigating solution, CHX(L) chlorhexidine liquid 2% (Arte & Vida, Bom Jesus de Itabapoana, RJ, Brazil); CHX(G) chlorhexidine gel 2% (Arte & Vida, Bom Jesus de Itabapoana, RJ, Brazil); NaOCl(L) sodium hypochlorite liquid 5,25% (Fórmula & Ação, SP, Brazil); NaOCl(G) sodium hypochlorite gel 3% (Fórmula & Ação, SP, Brazil); NAT natrosol gel (Fórmula & Ação, SP, Brazil) (control group), LO lubricating oil WD-40 (Ap Winner Ind, Ponta Grossa, PR, Brazil) (control group).

The files were inspected for deformities at high magnification (13.6 × ) (Zeiss Pico; Carl Zeiss MediTec, Dublin, California, United States), and none of them was discarded. Noncorrosive stainless-steel blocks against NaOCl or CHX were used to test resistance to dynamic cyclic fracture. The artificial canal was 1.5 mm wide, 20 mm long, and 3.5 mm depth, with a straight cervical segment of 14.29 mm, a long curved apical segment of 4.71 mm with a radius of 3 mm and a curvature of 90 degrees, and a long straight apical segment of 1 mm. 19

These dimensions allow the file to rotate freely within the artificial canal, both angularly and in a dynamic motion. The canal was covered with an acrylic plate to prevent instrument slippage, visualize the instrument during its action, and keep the irrigating solution within the simulated canal. The interface between the metallic block and the acrylic plate around the metallic canal was sealed with silicon (Pulvitec Polystic, São Paulo, SP, Brazil) to prevent leakage and keep the simulated canal full of irrigating solution. The irrigating solution was inserted into the simulated canal by using a 3-mL syringe (Ultradent Products Inc – EUA) and needle Navitip 30G 21mm long (Ultradent Products Inc – EUA).

The stainless-steel block with the artificial canal was positioned vertically on a heating plate (Fisatom, São Paulo, SP, Brazil) at a constant temperature (37°C ± 1°C), measured by a laser thermometer pointed at the simulated canal (MT-320 Minipa, Joinville, SC, Brazil), so the substance temperature into the root canal was accurately measured.

The contra-angle handpiece was fixed to the mechanical system that enables a dynamic axial movement of the file inside the simulated canal. The mechanical motion system consists of a linear guide, coupled with a savox sc-12 56t69 engine (Savox, Taichung, Taiwan) that performs back and forth movements, controlled by an electronic device that controls the speed amplitude of the axial movement. The files were placed inside the simulated canal coupled to a 6:1 reduction handpiece (Sirona Dental Systems GmbH, Bensheim, Germany), driven by a VDW Silver Reciproc motor (VDW, Munich, Germany). Cyclic fatigue tests were performed by rotating the instruments in continuous rotation at 800 rpm and torque of 1 Ncm.

The instruments were inserted 20 mm into the canal, fitted with a silicone stop to register this length. A back-and-forth axial movement at a speed of 3.0 mm/s and amplitude of 3.0 mm were applied to the instruments to simulate clinical pecking motion. The continuous rotation of the file's movement occurred until the fracture could be visually observed. The file movement within the simulated canal was recorded with iPhone 6s (Apple Inc. Cupertino, California, United States), using 4K recording technology. The movie was analyzed in Microsoft Movie Maker (Redmond, Washington, United States), and the time at which the instrument began to rotate until the moment of the fracture was registered in seconds. The number of cycles to fracture (NCF) was calculated by using the following formula: NCF = time (seconds) × revolution per minute/60.

The fragment lengths were measured by using a 150-mm digital caliper (accuracy of ± 0.03 mm/0.001). The maximum point of stress in both artificial canals was also analyzed.

## Statistical Analysis

A descriptive analysis of the NCF was performed according to the irrigant type (CHX and NaOCl) in its different formulations (G or L) and according to the control groups (LO and NAT). The normality and homoscedasticity were confirmed by the Shapiro–Wilk and Levene tests, respectively. Tukey's test was used to compare the averages for the NCF according to the irrigant type (groups = 6). The significance level was set at 5%.

## Results

The CHX(G), CHX(L), and OIL (LO) groups showed no significant difference between them and presented longer time and NCF. NaOCl(L) shows the NCF without significant differences between NaOCl(G) and NAT. The NCF of the NaOCl(G) was statistically similar to the CHX(L) and statistically lower than the CHX(G) and OIL groups. NAT did not present a statistical difference of the NaOCl(L) and NaOCl(G), and presented a significantly NCF than CHX(G). The irrigating agents did not significantly influence the length of the file fragment ( *p* = 0.066; Table 1 ).

**Table 1:** Means and standard deviations of number of fracture cycles and length of file fragments according to each type of irrigating substance

Irrigating solution	NCF Mean (SD)	Fragments (mm) Mean (SD)
CHX gel 2%	1,761 (258) ^a^	4.36 (0.20) ^A^
CHX solution 2%	1,523 (327) ^a,b,c^	4.30 (0.20) ^A^
NaOCl solution 5.25%	1,028 (389) ^d^	4.51 (0.29) ^A^
NaOCl gel 3%	1,111 (312) ^c,d^	4.25 (0.21) ^A^
Natrosol gel	1,309 (170) ^b,c,d^	4.27 (0.16) ^A^
Lubricating oil	1,613 (468) ^a,b^	4.42 (0.19) ^A^
*p* -Value	*p* < 0.001	*p* = 0.066

Note: Different lowercase letters in the columns indicate statistical difference ( *p* < 0.05). Same lowercase letters in the columns indicate that there was no statistical difference ( *p* > 0.05).

## Discussion

The experiment was conducted in a noncorrosive stainless-steel block to ensure the experiments' standardization without influencing other variables. 20 Because of higher cyclic fatigue resistance than that of the 30/0.04 Ni-Ti rotary instruments immersed in water at simulated body temperature, 15 XPS instruments were the ideal instrument in this study. The files were subjected to back-and-forth axial movements at a 3.0 mm/s 21 speed to simulate clinical conditions. In the dynamic model, 22 tensile and compressive stresses are distributed over a wider area throughout the instrument shaft within the artificial canal curvature by moving the file axially, 23 enhancing fracture resistance, and could reproduce a clinical up-and-down motion. 24

The radius and the angle of canal curvature are known to have significant roles in cyclic fatigue failure; lower degrees of curvature will result in longer fracture time. 25 In the present study, a severe 90-degree curvature of the simulated canal was used to assess the instrument's behavior in critical conditions. 19

Synthetic oil is a universal substance used to control cyclic fatigue resistance tests in a static or dynamic model. 26 The natrosol gel (hydroxyethyl cellulose) was also used as a control substance because it is the base of the CHX(G) and a nonionic agent, highly inert and soluble in water, 27 similar to the gel used in NaOCl(G). However, the manufacturer does not disclose the gel substance used in NaOCl(G).

The present study evaluated the instruments at 37°C simulating body temperature (13), whereas previous studies evaluated at room temperature. 28 29

Despite NaOCl is potentially irritating for periapical tissues, especially in high concentrations, it is the substance most widely used for root canal irrigation in endodontics because of its effective antimicrobial activity and ability to dissolve organic tissues. 30 Furthermore, NaOCl gel has been studied in the efficacy against microorganisms 12 and in the debris extrusion during endodontic treatment 11 with promising results.

On the other hand, CHX (L and G formulation) was used in the present study because both CHX and NaOCl were equally effective in reducing endodontic infection, despite their different molecular mechanisms. 8 The main limitation of CHX as an endodontic irrigator is its inability to dissolve pulp tissue. 24

In this study, OIL(LO), CHX(L), and CHX(G) did not differ significantly from each other; however, the NCF were statistically higher compared to NaOCl(L) ( *p* < 0.01). Therefore, the null hypothesis was rejected, corroborating with another study. 19 This result occurred probably due to deteriorations caused by NaOCl(L) on the surface of the file 31 and due to a galvanic reaction when the file is exposed to an electrolytic solution such as NaOCl(L), causing corrosion processes predisposing the instruments to unexpected fractures. 32 In addition, NaOCl(L) may cause micropitting by removing nickel from the instrument surface, 33 thereby decreasing the resistance to cyclic fatigue. 34

On the other side, CHX(G) provided the endodontic file the most significant resistance to fracture in this experimental model similar to CHX(L). Chlorhexidine gluconate is a cationic biguanide used as an intracanal irrigant in a liquid 32 and gel 15 formulation. The gel base used in the CHX(G) formulation in the present study was the natrosol gel (hydroxyethyl cellulose), which is a nonionic and inert and water-soluble agent 27 widely used to thicken shampoos, gels, and soaps based on cationic substances such as chlorhexidine gluconate. The cationic chlorhexidine molecule could avoid galvanic current preventing corrosion of the file metal decreases instrument breakage. 15 In the present study, CHX(G) had no differences regarding NAT, showing that CHX(G) increases the resistance to file fracture more by avoiding galvanic corrosion than being a lubricant.

In the NaOCl(G) group, the time to fracture and the NCF was similar to the one verified with the use of CHX(L) and NAT but did not reach the time until fracture and NCF verified with the OIL and CHX(G). When exposed to a NaOCl, even in a G formulation, the lower fracture resistance could be attributed to the induced corrosive zones, which are likely to reduce the resistance to cyclic fatigue of the instrument. 35 Besides, when exposed to a higher NaOCl concentration, a decrease in cyclic fatigue resistance is expected due to the increased amount of available chlorine that attacks the metallic league. 36 NaOCl, when compared with water, negatively affects the fatigue resistance of NiTi instruments, 37 especially at higher concentrations. 38

Immersion of instruments in NaOCl before cyclic fatigue testing for 3 to 5 minutes did not affect the cyclic fatigue of NiTi endodontic instruments. 39 However, they do not reflect the actual clinical status of root canal preparation in that they are performed in the presence of irrigants in the root canal. The current study kept the simulated canal full of irrigants throughout the experiment, better simulating the clinical conditions. Fatigue failure can be caused initially by cracks on the instrument's surface, which may be due to the concentration of chloride ions in the corrosion reaction under a titanium gap, 40 influencing instrument fatigue resistance. 41

The mean lengths of fractured segments were recorded to evaluate the maximum concentration area of the compression and tensile stresses of the tested files inside the canal curvature. There was no significant difference between the groups, regardless of the broken fragments' lengths (4.5 ± 0.5 mm). This matches the file's location inside the curvature; the point of maximum stress was similar in each circumstance, suggesting standardization of the experiment. 26

Based on the current study's findings, it is possible to conclude that CHX (G) and CHX (L) increased the time to fracture and the NCF of XPS files. In addition, XPS instruments were significantly more resistant to cyclic fatigue when irrigated with CHX(G) than with NaOCl(G), NaOCl(L), or CHX(L). However, the circumstances tested in these dynamic models are very different from those present in clinical practice. Therefore, further studies are needed to evaluate other irrigating solutions and their influence on XPS instruments' cyclic fatigue.
